# Stimulation Mapping Using Stereoelectroencephalography: Current and Future Directions

**DOI:** 10.3389/fneur.2020.00320

**Published:** 2020-05-12

**Authors:** Derek D. George, Steven G. Ojemann, Cornelia Drees, John A. Thompson

**Affiliations:** ^1^School of Medicine, University of Colorado School of Medicine, Aurora, CO, United States; ^2^Department of Neurosurgery, University of Colorado School of Medicine, Aurora, CO, United States; ^3^Department of Neurology, University of Colorado School of Medicine, Aurora, CO, United States

**Keywords:** epilepsy, stereoelectroencephalography, electrical stimulation mapping, epileptogenic zone, subdural grid, review, functional neurosurgery

## Abstract

Electrical stimulation mapping (ESM) using stereoelectroencephalography (SEEG) is an essential component in the workup of surgical epilepsy. Since the initial application of ESM in the mid-1960s, it remains unparalleled in defining eloquent brain areas and delimiting seizure foci for the purposes of surgical planning. Here, we briefly review the current state of SEEG stimulation, with a focus on the techniques used for identifying the epileptogenic zone and eloquent cortex. We also summarize clinical data on the efficacy of SEEG stimulation in surgical outcomes and functional mapping. Finally, we briefly highlight future applications of SEEG ESM, including novel functional mapping approaches, identifying rare seizure semiologies, neurophysiologic investigations for understanding cognitive function, and its role in SEEG-guided radiofrequency thermal coagulation.

## Introduction

Electrical stimulation mapping (ESM) is a vital component of the workup for epilepsy surgery, allowing for the determination of the functional cortex and helping to localize the epileptic network and its functional impact (1). The two conventional extraoperative invasive methods for obtaining this information are stereoelectroencephalography (SEEG) and subdural grids (SD). While both SEEG and SD ESM are appropriate techniques, and the decision to use one vs. the other is highly individualized to each patient, our review focuses on SEEG ESM. Also, it is not uncommon for local expertise and availability of these surgical services, at the treating center, to contribute to the preferential use of either SEEG or SD for extraoperative intracranial monitoring. This review aims to provide insights into (1) the current practice and clinical decision-making for the application of SEEG stimulation mapping; (2) clinical data and outcomes associated with this technique; and (3) future applications of SEEG stimulation mapping for clinical practice and basic neuroscientific investigation.

The technique of SEEG ESM originated from the pioneers of SEEG itself, neurosurgeon Jean Talairach and neurologist Jean Bancaud. During their time together at Hôpital Sainte Anne in Paris, France, they improved the methods used for the stereotactic implantation of electroencephalography (EEG) leads. They also defined the concept of the anatomico-electrico-clinical nature of the epileptogenic zone (EZ), proposing that the EZ is organized as a network with unique anatomic correlates, electrographic properties, and clinical manifestations (2, 3). Talairach and Bancaud started their investigations using SEEG stimulation during the mid-1960s, looking at motor responses from cingulate stimulation (4) and other mesial frontal lobe areas (5). SEEG ESM is therefore not a novel technique and has been utilized by French and other European centers for decades in the workup of surgical epilepsy (6–9). However, in the last decade, increased use of SEEG in the workup of surgical epilepsy by many North American centers has been matched by a parallel increased interest in the use of ESM as a complementary tool to SEEG recordings (10).

### Current Practice and Decision-Making for the Use of SEEG Stimulation Mapping

**Figure 1:**
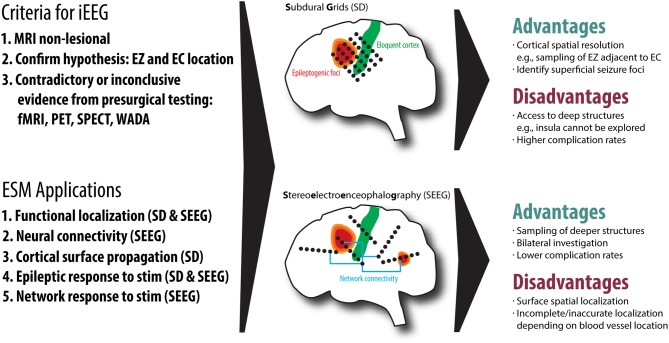
An overview of the two common invasive EEG (iEEG) techniques, subdural grids (SD) and Stereoelectroencephalography (SEEG), including the criteria for iEEG, electrical stimulation mapping (ESM) applications, advantages and disadvantages of both techniques. We also demonstrate the relationship of the epileptogenic zone (EZ) to eloquent cortex (EC), highlighting that SEEG is useful for understanding network connectivity.

ESM *via* SEEG is becoming an increasingly popular technique for localizing the EZ and eloquent cortex (EC) in patients with epilepsy requiring surgery. The decision to use SEEG mapping always begins with considering whether the patient requires invasive EEG (iEEG) monitoring. In general, iEEG is considered when noninvasive testing has not unequivocally identified the EZ. Very often, this is the case when magnetic resonance imaging (MRI) is non-lesional; that is, MRI has not revealed an obvious pathology capable of producing seizures (e.g., mesial temporal sclerosis, encephalomalacia, gray matter heterotopias, focal cortical dysplasia, polymicrogyria, low-grade neuroepithelial tumors, vascular malformations, or other structural lesions). The process follows the established expert opinion on the use of iEEG for extraoperative monitoring of epilepsy patients (11). Before iEEG, a reasonable hypothesis about the location of the EZ and its relationship with EC must exist, and clinical data from noninvasive diagnostic tests [e.g., scalp EEG, functional MRI (fMRI), positron emission tomography (PET), single-photon emission computed tomography (SPECT), and sodium amytal (Wada) testing] show contradictory or inconclusive evidence of EZ localization (10, 12, 13). If removal is considered, EZ must be in an operable location, although iEEG data can also be used to place targeted intracranial responsive neurostimulation devices (RNS®). Additional considerations for the location of the EZ are important: a cortical area easily accessed *via* craniotomy may be more easily recorded from and stimulated using intraoperative electrocorticography (ECoG) or extraoperative subdural grids, whereas SEEG may provide more precise information for a deeper cortical area, such as the medial cortex. In cases with suspected insular seizure onset, SEEG is mandatory as subdural grid placement is not feasible. See Figure 1 for an overview of the decision-making of SEEG vs. SD.

The safety, morbidity, and mortality of iEEG modalities must also be considered. Because SEEG electrodes perforate the dura and penetrate into the brain, intracranial trajectory planning accounting for collisions with cerebral vasculature must be considered, and a multidisciplinary team must be used to assess the placement of electrodes along most likely pathways of electrical spread (10). Assuming these safety considerations are met, the complication rates from SEEG have been shown to be generally low: Mullin et al. reported a pooled complication prevalence of 1.3% (hemorrhage, 1%; infection, 0.8%; electrode malpositioning, 0.6%; hardware malfunction, 0.4%; and death, 0.3%) (14), and Cardinale et al. reported an overall complication prevalence of 1.8% [three major complications (0.4%) and one fatality (0.1%)] (9). Additionally, SEEG has been shown to have lower morbidity and higher tolerability by patients than subdural electrodes or intraoperative ECoG (13, 15). Beyond the safety considerations, additional factors related to the electrical stimulation of neural tissue must be addressed, namely, charge density and charge per phase (1). While both animal (16) and human (17) studies have investigated these parameters, and a charge density safety threshold of 30 μC/cm^2^ has been proposed (18), no consensus exists regarding safe stimulation parameters. Furthermore, SEEG electrodes are manufactured with varying diameters (range, 0.80–0.86 mm), contact number (range, 4–18), and contact length (range, 2.0–2.5 mm), further influencing charge density safety considerations. Standardization of the stimulation parameters and electrode characteristics may therefore be beneficial.

Following implantation, a multiday in-patient observation proceeds to capture habitual seizures. To increase the likelihood that a patient will experience a spontaneous seizure and exhibit associated ictal semiology while under observation, anti-epileptic drugs (AEDs) are often discontinued. However, cortical stimulation is typically—and more safely—performed after anticonvulsants have been restarted. The reasons for this are 2-fold: AEDs decrease the likelihood of generating non-habitual seizures or after-discharges, thus making stimulation safer for the patient and increasing confidence in the results of stimulation. ESM often occurs prior to hardware explantation at the end of the observation period, after AEDs have been restarted. Once the decision is made to use stimulation to locate EC and help identify the EZ, the next step is deciding the appropriate stimulation protocol. Currently, no standardized stimulation protocols exist. Various institutions have developed different protocols for SEEG ESM for reproducing habitual seizure semiology [see Table 1 from (10)]. The newest French guidelines on SEEG state that bipolar and biphasic current should be used between two contiguous contacts to stimulate the target of interest. The guidelines also suggest parameters for both low- and high-frequency stimulation, as found in Table 1.

**Table 1:** SEEG stimulation parameters as outlined by the French guidelines on SEEG (19).

**Stimulation paradigm**	**Frequency (Hz)**	**Pulse width (ms)**	**Pulse intensity (mA)**	**Stimulation duration (s)**
Low frequency	1	0.5–3	0.5–4	20–60
High frequency	50	0.5–1	0.5–5	3–8

The proposed paradigm of low- and high-frequency stimulation is useful in targeting specific cortical regions based on that region's after-discharge threshold. For example, the Heschl gyrus, motor cortex, the hippocampus, and dysplastic cortex are frequent targets of periodic low-frequency stimulation (1 Hz, 0.5–5 mA, 0.3–0.5 ms) because of their lower after-discharge threshold, while higher-frequency stimulation (50 Hz, 0.5–3 mA, 0.5 s, 1–5 s) is used to stimulate all other areas (20). A widely adopted approach for mapping functional connectivity, initially established by Valentin and others (21), uses low-frequency (0.2–1 Hz), single-pulse electrical stimulation (SPES) (21, 22). The sub-hertz low-frequency stimulation used in SPES elicits electro-epileptiform activity without inducing a clinical response (23). This protocol has been applied to several epileptic pathologies, including type I and II cortical dysplasia, hippocampal sclerosis, and polymicrogyria (23). Independent of the tissue characteristics, after-discharge threshold is also influenced by anticonvulsant levels and the amount and frequency of preceding seizures.

Apart from spontaneously occurring habitual seizures, the interpretation of post-stimulation EEG provides insight into the localization of the EZ. As it is hypothesized that the EZ is structured as a neural network with cortical areas connected via white matter pathways, stimulation-induced synchronization of this network can support proper localization. Combining the anatomic locations of stimulated regions, stimulation-induced ictal patterns and spontaneous ictal onset and spread with observed semiology provide data on the organization of EZ networks. Ideally, seizure semiology should follow EZ stimulation if the EZ is close to the symptomatogenic cortex and stimulated with sufficient current. Otherwise, the assumption is that the EZ is not being properly sampled (24). One more complicating factor in this paradigm is the fact that some epilepsy types (temporal plus and posterior quadrant epilepsies) may require stimulation at more than one area of the network to induce a seizure. The failure to induce a clinical seizure may therefore not be because of suboptimal contact placement but rather because of insufficient network stimulation (24). Additionally, stimulation of distant areas connected to the EZ may induce a seizure, making seizure production *per se* less important than the combined anatomic, electrographic, and clinical data.

While SEEG ESM provides advantages for pinpointing EZ and EC, the technique has several important limitations. First and foremost, the efficacy of SEEG ESM for isolating EZ and EC depends upon appropriate electrode placement. Inappropriate electrode placement can lead to inadequate stimulation of the EZ, which can result in an inappropriate hypothesis of the EZ location. Even if appropriately placed, SEEG can offer lower 3D spatial resolution than SD ESM as SEEG electrodes are often placed several centimeters away from one another, whereas subdural grid electrodes are separated by millimeters (25). Different cortical regions require specific stimulation paradigms for seizure generation. Inadequate stimulation—even with properly placed electrodes—may therefore lead to false-negative or false-positive results. And while depth electrodes may be placed orthogonal to cortical surfaces—especially the insular cortex (26–29)—they are often positioned perpendicular to the cortex, which may limit the technique's utility in differentiating adjacent eloquent and non-eloquent areas (25, 28). Given the limitations of SEEG and SD and the lack of comparative studies on SEEG and SD ESM, the functional mapping strategy must be individualized for each patient.

### Primary Clinical Outcome Metrics Derived From SEEG Stimulation Mapping

The primary clinical outcome measures of SEEG stimulation relate to its ability to delineate the EZ, differentiate the EZ from the EC, and ultimately its contributions to seizure freedom. To date, few studies have directly correlated the use of SEEG ESM with surgical outcomes as measured using the Engel classification system. However, more studies have investigated the ability of stimulation to induce electro-clinical seizures matching the patient's spontaneous (i.e., habitual) seizures. This so-called concordance of stimulation-induced seizures with spontaneous seizures provides additional data on the probable location of the EZ. The studies reporting data on the concordance of ESM with spontaneous seizures have investigated multiple types of focal epilepsy, including frontal lobe epilepsy (FLE) (30–34), temporal lobe epilepsy (TLE) (30–35), parieto-occipital epilepsy (POE) (30, 32, 33), and multifocal epilepsy (32, 33), or special circumstances such as epilepsy arising from hypothalamic hamartomas (36). Of note is that the study from Chassoux investigated EZ localization within the specific context of focal cortical dysplasia and the study from Chauvel et al. only selected for patients with concordant seizures. Concordance rates have varied from 26% (lateral temporal lobe) (31) to 100% in areas of focal cortical dysplasia (33). Of the studies listed, only Bernier et al. commented on surgical outcomes. In their retrospective analysis of concordance data from patients undergoing resection with Engel class I and II outcomes at 1 year, patients undergoing unilateral temporal stimulation showed 100% (19/19) concordance for side and 95% (18/19) concordance for site (30). Recently, Oderiz et al. published a retrospective study investigating the contribution of SEEG cortical stimulation to surgical outcomes. They showed that the proportion of patients with stimulation-induced electro-clinical seizures was higher in the good outcome group (Engel class I at 42.2-month median follow-up) than the bad outcome group [Engel class II or greater; 31 of 44 (70.5%) vs. 28 of 59 (47.5%), *P* = 0.02] (37). The group concluded that stimulation-induced seizures are just as reliable as spontaneous seizures in localizing the EZ in focal epilepsy, assuming appropriate placement and stimulation of the target tissue (37). Another study of patients with polymicrogyria (PMG) showed that, while only partial PMG-EZ concordance was found in 74% of cases, subsequent surgery resulted in a favorable outcome in 72% of patients (Engel class I at mean 4.6-year follow-up) (38). This group concluded that SEEG—including cortical stimulation—is warranted in the workup of PMG to define, if only partially, the EZ. While these studies suggest a benefit to seizure outcome using SEEG ESM, additional, multi-institutional retrospective and prospective studies are needed to further define the contributions of SEEG ESM to surgical outcomes.

The goal of functional mapping using SEEG is to identify EC and its relationship to the EZ. Such investigations are typically tailored to the individual patient based on the hypothesized location and functional connectivity of the EZ, and depend on where the electrodes are placed. Typical areas investigated in this fashion include sensory cortices (e.g., necessary somatosensory, visual, and auditory cortex), motor cortex, and language areas (20). Due to the frequent semiologic overlap with symptomatogenic cortical regions, sensory and motor cortices are relatively common targets, while language mapping proves to be more difficult due to the widely and individually distributed networks involved in language functions (20, 39). However, with contacts traversing through cortical regions as well as adjacent and intervening white matter, the results of cortical stimulation mapping may be difficult to interpret regardless of the area stimulated due to the heterogeneity of the elicited responses (40). For further details on the specifics of language mapping, see Trébuchon and Chauvel (20). One case study of extraoperative SEEG language mapping vs. intraoperative mapping showed discordant results (41). In a more recent case series, Young et al. demonstrated that SEEG may be equally as efficacious as ECoG in mapping language areas among a small cohort of 15 patients, resulting in safe resection or ablation in six cases (42). These findings are part of a small number of studies investigating the efficacy of SEEG ESM of language functions. Given the dearth of studies providing efficacy data, further investigations are needed to better understand the contribution of SEEG ESM to language mapping.

A few studies have looked at the contribution of SEEG functional mapping to surgical outcomes. In one study from Thorsteinsdottir et al., extraoperative high-frequency SEEG stimulation (biphasic, 50 Hz) paired with intraoperative stimulation helped isolate EC in 70 patients undergoing resection for focal epilepsy. Thirty percent (21/70) exhibited overlap between the EZ and EC. If EZ–EC overlap restricted total EZ resection, the resection was classified as incomplete. This notwithstanding, subsequent analysis revealed no statistically significant difference in the surgical outcomes between the eloquent and non-eloquent groups, with a median follow-up period of 31.5 months (eloquent group: 86% Engel class I; non-eloquent group: 82% Engel class I; *P* = 0.20) (29). Surgical morbidity included transient and permanent resection-related neurologic deficit in five patients and one patient, respectively (29). Interestingly, in a study assessing outcomes from bilateral lead implantation, the overlap of EZ with EC was associated with an increased seizure recurrence in patients undergoing surgical resection with mean follow-up of 3.1 years (range = 1–7.4, *P* = 0.04, OR = 2.16, 95% CI = 1.04–4.19) (43). These studies are seemingly contradictory. However, presumably in some cases of EZ–EC overlap, EZ resection is restricted to preserve EC. This leaves more of the EZ network intact and potentially reduces the chances of seizure freedom. Conversely, in other cases—in the hands of a more aggressive surgeon—the eloquent cortex is removed, a neurological deficit tolerated, and the likelihood for seizure freedom increased due to complete EZ resection.

### Future Clinical and Basic Science Applications of SEEG Mapping

Many unknowns and future possibilities exist for SEEG stimulation mapping. SEEG mapping has the potential to elucidate both basic neuroscientific and clinical knowledge as our understanding and application of this technology grows. The tool will also take on an important role in facilitating ablative procedures using radiofrequency thermal coagulation.

Further work with this technology, using both animal models and consented patients, will provide answers on technical questions about SEEG stimulation itself. One such area of research would include better defining the electrophysical properties most conducive to stimulation mapping, such as the biophysical properties of the cortical regions or the optimal charge density to minimize tissue damage (1, 44). Other investigations into optimal stimulation paradigms and excitation properties of certain cortical or subcortical areas could benefit our understanding of both normal regional physiology and pathologic excitability (45–48). Such investigations could include high-/low-frequency stimulation paradigms or rely on SPES (49–52). Improvements in SEEG lead construction could be undertaken to improve the stimulation efficacy and recording capabilities, such as with hybrid electrodes (53, 54). Cross-modality investigations utilizing SEEG combined with fMRI or magnetoencephalography (MEG) could be helpful in further elucidating the basic functions and functional connectivity of various brain regions (55).

The use of SEEG ESM for functional mapping has provided insights into the distinct function of deeper brain regions and their functional connections. The use of SEEG stimulation for these purposes will continue to expand. Recent investigations with human subjects have looked at the function of the prefrontal cortex (56), the frontal operculum (57), medial frontal lobe (47), cingulate cortex (58, 59), the fusiform gyrus (60–62), the insula (63, 64), the amygdala (65, 66), parietal cortex (67), the anterior thalamus (68), the pulvinar (69), and the claustrum (70). Other investigations have looked at multiple brain regions simultaneously, such as when investigating sleep (71, 72). These studies provided significant insights into the function of these brain regions and their relationship to epilepsy pathology, either through stimulation-induced behavioral changes, induction of novel subjective phenomena, recapitulation of rare seizure semiologies, or through investigation of cortical dynamics. Their findings have implications for future research into the normal functions of brain regions, organization and dynamics of functional networks, seizure semiologies, and pathologic network changes.

Besides anatomic and physiologic investigations, SEEG ESM will also play an essential role in treatment using SEEG-guided radiofrequency thermal coagulation (SEEG RF-TC). Bourdillon et al. and Isnard et al. have published the only recommendations on the topic. They suggest induction of habitual semiology with bipolar low- and/or high-frequency stimulation (3 mA for low frequency, 1 mA for high frequency) between two adjacent contacts to identify the potential EZ and subsequent target of coagulation (19, 73). Stimulation is also used to identify eloquent areas which may not be suitable for coagulation or areas which may be targeted if a minor neurologic deficit is to be tolerated (74, 75). A recent meta-analysis of SEEG RF-TC for focal epilepsy showed a pooled rate of permanent neurologic deficit of 2.5% (95% CI = 1.2–5.3%) (76). Of the five neurologic deficits observed at 1-year follow-up occurring in three studies, only one was unexpected, while the other four were expected and tolerated as the target tissue was in the primary motor cortex (76). SEEG ESM therefore plays an essential role in the safe implementation of SEEG RF-TC. The contribution of SEEG ESM to seizure outcomes following SEEG RF-TC is less clear, however. The meta-analysis from Bourdillon et al. showed a pooled seizure-free rate at 1 year of 23% (95% CI = 8–50%) and a pooled responder rate of 58% (95% CI = 36–77%) (76). However, the included studies suffered from significant heterogeneity, which precluded any strong recommendations on the use of RF-TC for the treatment of focal epilepsy. The exact contribution of SEEG ESM to seizure outcome after RF-TC is therefore unknown, and future investigations should aim to better define how SEEG ESM contributes to seizure outcome after RF-TC.

## Conclusion

SEEG cortical stimulation mapping is a technology with much promise in helping to isolate the EZ and define EC during the workup of surgical epilepsy. While there is a relative dearth of studies specifically investigating the contributions of stimulation to surgical outcomes, multiple studies have provided promising data for the technology's utility in refining pre-surgical planning based on the known extent of the EZ and overlap with EC. ESM is a crucial part of invasive monitoring, and future studies should aim to better define optimal stimulation parameters and the specific contribution of ESM to surgical outcomes. Moreover, SEEG stimulation has been proven, and will continue, to be a valuable tool in treating focal epilepsy *via* RF-TC, elucidating the function and connectivity of deeper brain regions and providing novel insights into brain physiology and pathophysiology.

## Author Contributions

SO, JT, and CD contributed to the conception and design of the manuscript. DG conducted the literature search and wrote the first draft of the manuscript. DG, JT, and CD produced the figure and table. All authors contributed to manuscript revision and read and approved the submitted version.

## Conflict of Interest

The authors declare that the research was conducted in the absence of any commercial or financial relationships that could be construed as a potential conflict of interest.
